# Hepatocellular Carcinoma in Cirrhotic Versus Noncirrhotic Livers: Clinicomorphologic Findings and Prognostic Factors

**DOI:** 10.5152/tjg.2023.21791

**Published:** 2023-03-01

**Authors:** Ümit Karaoğullarından, Oğuz Üsküdar, Emre Odabaş, Numan Ak, Sedef Kuran

**Affiliations:** Department of Gastroenterology, Çukurova University, Adana, Turkey

**Keywords:** Cirrhossis, HCC, non-cirrhossis, survival

## Abstract

**Background:**

Hepatocellular carcinoma mostly develops in a cirrhotic (80%) background. The clinical features of cirrhotic hepatocellular carcinoma and non-cirrhotic hepatocellular carcinoma also differ. We aimed to determine the clinicopathologic features, tumor characteristics, treatment options, and overall survival after diagnosing hepatocellular carcinoma and prognostic factors effective on survival of hepatocellular carcinoma developing in cirrhotic and non-cirrhotic conditions.

**Methods:**

In our study, 220 patients aged over 18 years who were histologically diagnosed as having hepatocellular carcinoma were included. The patients were divided into 2 groups as cirrhotic and non-cirrhotic.

**Results:**

When the tumor morphologies were examined in our study, it was observed that they were mostly solitary in both groups. Cirrhotic hepatocellular carcinomas had significantly higher rates of invasion than the non-cirrhotic group (35.3% vs. 20.3%, respectively) (*P*  < .05). The survival rate was found to be better in the non-cirrhotic group (17.5 months vs. 11.5 months) (*P*  < .05). Age, maximal tumor diameter, and morphologically infiltrative tumor character were found to be independent risk factors affecting survival in patients with cirrhosis. Portal vein invasion, alfa-fetoprotein, and the absence of an underlying risk factor in the etiology were observed as independent risk factors affecting survival in patients with non-cirrhosis.

**Conclusion:**

Cirrhotic hepatocellular carcinoma and non-cirrhotic hepatocellular carcinoma had different clinicopathologic features and risk factors. We analyzed that treatment choice trends were different between the 2 groups. We also observed that the factors that affected survival were different between the 2 groups.

**Main Points**

- The aim is to reveal the clinical and etiological differences in cirrhotic and non-cirrhotic patients and reveal tumor morphological differences in cirrhotic and non-cirrhotic patients.
- Cirrhotic hepatocellular carcinomas had significantly higher rates of invasion than the non-cirrhotic group (35.3% vs. 20.3%, respectively) (P < .05).
- It was analyzed that treatment choice trends and the factors that affected survival were different between the 2 groups.

## Introduction

Hepatocellular carcinoma (HCC) is the sixth most common cancer in the world and the second most common cause of cancer-related death.^1^ Hepatocellular carcinoma accounts for more than 90% of all primary liver cancers.^2^ Hepatocellular carcinoma mostly develops in a cirrhotic (80%) background and is called cirrhotic-HCC (CHCC). Hepatocellular carcinoma develops less frequently in a non-cirrhotic (20%) background and is called non-cirrhotic-HCC (NCHCC).^3-7^ The clinical features of CHCC and NCHCC also differ because of the different mechanisms leading to HCC.

The main risk factors for the development of HCC can be listed as hepatitis B virus (HBV), hepatitis C virus (HCV), delta virus (HDV), long-term excessive alcohol consumption, obesity, and aflatoxin B1.^8,9^

The prognostic prediction and appropriate treatment options in patients with HCC are often complex because patients frequently have cirrhosis and additional comorbidities.^10^ The patient’s tumor burden, general performance, and the degree of underlying liver failure are very effective in determining treatment.^11,12^

The severity of the underlying liver disease has a major impact on treatment decisions and prognosis in patients with HCC. The presence of cirrhosis and consequent deterioration of liver function may limit surgical and non-surgical options. In contrast, the absence of cirrhosis may favor the use of curative surgical treatment.^13^

In our study, we aimed to determine the clinicopathologic features, tumor characteristics, treatment options, and overall survival after diagnosing HCC, and prognostic factors effective on survival of HCC developing in cirrhotic and non-cirrhotic conditions.

## Materials and Methods

In our study, 220 patients aged over 18 years who were histologically diagnosed as having HCC between 2016 and 2020 were included. Biopsies were taken for scientific study purposes from most of the patients diagnosed with HCC during dynamic magnetic resonance imaging (MRI) in our clinic. Biopsies were not taken from patients with coagulopathy, severe thrombocytopenia, and decompensated liver disease. Biopsies were obtained from approximately 80% of patients with radiologically diagnosed HCC. Data about the patients were obtained from the electronic information system. All follow-up of the patients was performed in a single center.

The patients were divided into 2 groups: CHCC and NCHCC. The distinction between CHCC and NCHCC was made based on the laboratory, radiologic, and/or histologic findings of cirrhosis. The diagnosis of cirrhosis was made with the combination of the Bonacini cirrhosis discriminant score [platelet (PLT), alanine aminotransferase/aspartate transaminase (AST) ratio, international normalized ratio (INR)] and radiologic findings.^14^ Radiologic findings were evaluated in terms of hepatic right lobe atrophy, left lobe and caudate lobe hypertrophy, heterogeneous and/or nodular appearance in the parenchyma, portal vein enlargement, portal vein flow evaluation, portal vein thrombosis, splenomegaly, and ascites. Patients with liver parenchyma biopsy were evaluated using METAVIR (meta-analysis of histological data in viral hepatitis) scoring. The METAVIR system scores fibrosis on a 5-point scale, with F0: no fibrosis, F2-F3: significant fibrosis, and F4: cirrhosis.^15^

Demographic data of the patients were analyzed.

Etiologic data were recorded, including HBV, HCV, hepatitis D virus (HBV+HDV), non-alcoholic fatty liver disease (NAFLD), alcohol-induced liver disease (ALD), and Cryptogenic.

Non-alcoholic fatty liver disease/non-alcoholic steatohepatitis diagnosis was determined by excluding other liver diseases and with alcohol intake <30 g/day in men and <20 g/day in women with histologic or radiologic signs of fat.^16,17^

Alcohol consumption for ALD was defined as an average of >210 g per week for men or >140 g per week for women over a period of at least 2 years.^18^

Tumor characteristics and morphologic features, maximal tumor diameter (MTD), number of lesions, portal vein invasion, and infiltrative character, were recorded.

Portal vein tumor invasion: It was considered positive on dynamic MRI in areas with portal involvement or in patients meeting other malignant criteria. Concurrently, these patients were considered malignant in patients with d-glucose uptake on positron emission tomography/computed tomography.^19-21^

We examined whether the patients received surgical treatment (resection, liver transplantation), locoregional treatment (ablation, transarterial chemoembolization [TACE], transarterial radioembolization [TARE]), and systemic treatment or supportive treatment.

Patient’s survival times were evaluated from the time of HCC diagnosis.

This study was conducted in accordance with the ethical rules of the Declaration of Helsinki. The related study was approved by Cukurova University Ethics Committee (113/2021).

### Statistical Analysis

The normality of data distribution of continuous variables was examined using the Shapiro–Wilk test. The Mann–Whitney *U*-test was used for comparisons made according to the cirrhosis groups because the variables did not conform to normal distribution. In the analysis of categorical variables, the Chi-square test and Fisher’s exact test were used. Univariate and multiple Cox regression models were created to investigate factors that affected life expectancy. The data analysis was performed using the Statistical Package for the Social Sciences software 21 program (IBM Corp.; Armonk, NY, USA).

## Results

The clinical features, etiologic data, laboratory findings, and overall survival after diagnosis of HCC of all patients are given in Table 1.

There was no difference between the CHCC and NCHCC groups in terms of age and sex distribution (*P*  > .05). Significant male dominance was observed in both groups. The male dominance rate was the same between the CHCC and NCHCC groups (86.2% and 87.8%, respectively).

Hepatitis B was the most common cause in both groups. Accordingly, HCC secondary to hepatitis C developed significantly in a cirrhotic background (85% in a cirrhotic background), and the rates of CHCC and NCHCC developing in the NAFLD background were almost the same (48.2% in a cirrhotic background, 51.8% in a non-cirrhotic background).

There was significant hepatitis B dominance in the NCHCC group (62.2%).

Aspartate transaminase, albumin, INR, AST to PLT ratio index, PLT, and total bilirubin values were significantly different between the 2 groups (*P*  < .001).

When the survival times were examined, it was observed that the survival time was borderline better in the NCHCC group (11.5 months vs. 17.5 months) (*P*  < .05).

Tumor morphologic features and treatment choices are given in Table 2. Tumor focality rates differed according to the groups (*P*  = .012). Accordingly, the infiltrative appearance of the tumor was higher in patients with cirrhosis (*P*  < .05).

Portal vein invasion was more common in the CHCC group (*P*  < .05).

There was no significant difference between the groups in MTD and alfa-fetoprotein (AFP) values (*P*  > .05).

When evaluated in terms of treatment choices, it was seen that resection and TARE were significantly preferred in the NCHCC group (*P*  < .001). More supportive treatment was given in the CHCC group (*P*  < .05).

The factors affecting survival in the CHCC group are given in Table 3. In univariate analysis, age, the presence of HCV in etiology, MTD, and portal vein invasion were found to be effective on survival. Age, MTD, and an infiltrative appearance of the tumor were observed as independent risk factors in multivariate analysis.

The factors affecting survival in the NCHCC group are given in Table 4. In the univariate analysis, cryptogenic, NAFLD, portal invasion, and AFP were found to be significant. In multivariate analysis, portal invasion, AFP, and cryptogenic in the etiology were observed as independent risk factors.

## Discussion

Hepatocellular carcinoma development was observed in 62.7% of the patients with a cirrhotic background and 37.3% with a non-cirrhotic background. In studies conducted in Western countries, an average of 20% NCHCC was observed^3-7^; the rate of HCC in non-cirrhotic backgrounds was higher in our study. This may be due to the changing etiologic factors in different geographies and the leading risk factors effective in hepatocarcinogenesis. This may also be related to the small number of our patients and the fact that we only included patients with biopsy-proven HCC in our study. There was etiologically significant HBV dominance (62.2%) in HCC that developed in non-cirrhotic backgrounds. It was observed that HCV led to the development of HCC in cirrhotic backgrounds. This may be due to the direct oncogenic effect of HBV. In the background of HCV, HCC may be associated mostly with the development of advanced fibrosis and cirrhosis.^22-25^

In our study, the survival rate was found to be better in the non-cirrhotic group (17.5 months vs. 11.5 months) (*P*  < .05). Studies have also shown that patients with NCHCC have a better life expectancy.^26-28^ However, there are studies in which there is no difference in life expectancy between the 2 groups.^29^

In our study, no significant difference was observed between the groups in terms of AFP levels (*P*  = .18). There were studies with significant AFP elevation in the CHCC group.^30^ In a review, significant AFP elevations were observed in the CHCC group.^31^ An AFP level of 20 ng/mL is a commonly used threshold value for HCC assessment in clinical practice.^30,32^ In our study, AFP >20 ng/mL was seen in 60.4% of all patients. Alfa-fetoprotein >20 ng/mL was observed in 62.3% of patients with cirrhosis. Alfa-fetoprotein >20 ng/mL was observed in 57.3% of the NCHCC group. In a study, AFP elevation was found in 70% of the patients.^33^

When the groups were compared in terms of MTD, MTD was greater in the NCHCC group, but no statistical significance was observed (*P*  = .22). This may be related to the lack of regular medical follow-up in the NCHCC group and the diagnosis of tumors when they were symptomatic. There are studies in which MTD is significantly higher in NCHCCs^26,30^

When evaluated in terms of portal invasion, CHCCs had significantly higher rates of invasion than the NCHCC group (35.3% vs. 20.3%, respectively) (*P*  < .05). Similarly, the portal invasion was observed more frequently in the CHCC group.^30^ In another study, it was shown that the risk increased in the CHCC group, especially as the Child–Pugh score increased.^31^

When the tumor morphologies were examined in our study, it was observed that they were mostly solitary in both groups. A tendency to be more solitary was observed in the NCHCC group (60.9%) than in the CHCC group (47.9%). Compared with the NCHCC group, infiltrative tumor appearance was significantly more common in the CHCC group. In a review, it was shown that it was multifocal in CHCCs and mostly solitary in NCHCCs.^34^ In an HBV-related study designed in an animal model, it was observed that tumors tended to be more solitary in both groups of HCC developing in cirrhotic backgrounds and HCCs developing in non-cirrhotic backgrounds, but this tendency was more dominant in the NCHCC group.^35^ In another study, the authors showed that CHCC had a more infiltrative appearance.^30^

Considering the treatment choices in our study, it was observed that hepatic resection was performed significantly more in the NCHCC group than in the CHCC group (*P*  < .01). The preference for resection may be related to the low risk of liver failure post-hepatectomy. Interestingly, although portal invasion was more common in the CHCC group, TARE was performed significantly more in the NCHCC group (*P*  < .01). In our study, although portal vein invasion was more common in the CHCC group, TARE was performed more frequently in the NCHCC group. This can be explained by the mean MTD of 7 cm (beyond TACE limits) in the NCHCC group. In patients who are at the border of TACE and TARE, the procedure may be referred to as TARE. At the same time, in patients who are suitable for TARE in the CHCC group, only supportive treatment may be given depending on the patient’s performance and the degree of the underlying disease. We could not find any studies in the literature about the frequency of TARE performance in CHCC and NCHCC. In the CHCC group, it was found that supportive care was more preferred compared with the NCHCC group (*P*  < .01). In a study, it was shown that resection was more frequently preferred in the NCHCC group and supportive care was preferred in the CHCC group, in correlation with our study. However, in contrast to our study, TACE was preferred more in the CHCC group.^28^ In another study comparing treatment choices, it was found that supportive care was more common in the CHCC group. Again, in the same study, it was seen that 2 locoregional treatments were more preferred in the CHCC group, without distinguishing between TARE and TACE.^26^

In our study, age, the presence of HCV in etiology, and MTD were found to be significant when the factors affecting survival in patients with cirrhosis were examined. Age, MTD, and morphologically infiltrative tumor character were found to be independent risk factors.

In our study, when the factors affecting life expectancy in the NCHCC group were examined, NAFLD, portal invasion, and AFP without risk factors were found to be significant in the etiology. Portal vein invasion, AFP, and the absence of an underlying risk factor in the etiology were observed as independent risk factors.

In a study conducted without distinction between CHCC and NCHCC, the presence of cirrhosis, age, maximal tumor diameter, and tumor multilobularity were seen as prognostic factors.^24^ Again, in another study conducted without distinction between CHCC and NCHCC, microvascular invasion and multilobular tumors were found to be prognostic factors.^36^ In an animal model and HBV-related study, MTD and multilobularity were shown as prognostic factors for NCHCC. In CHCC, however, female sex was seen as a prognostic factor.^35^

Our study had some limitations. Although all patients had HCC with a biopsy-proven diagnosis, the number of patients was limited. The study had a retrospective design. All patients were from a single tertiary care center. All diagnoses of patients in the NCHCC group could be made by taking a biopsy from normal non-tumor parenchyma.

## Conclusion

Cirrhotic-HCC and NCHCC had different clinicopathologic features and risk factors. Patients with NCHCC tended to have better liver reserve and more solitary tumors than patients with CHCC. We analyzed treatment choice trends, which were also different between the 2 groups. We observed that the factors that affected survival were different between the 2 groups. We found that survival was better in the NCHCC group. If supported by studies with larger patient numbers, distinguishing between patients with HCC with and without cirrhosis may help in the prognostic approach, diagnosis, and approach to treatment.

## Figures and Tables

**Table 1.** Demographic, Etiologic, and Laboratory Findings of the Patients

		Cirrhotic			Non-cirrhotic			*P*
		Mean ± SD	Median [IQR]	Min-Max	Mean ± SD	Median [IQR]	Min-Max	
Number of patients			138 (62.7%)			82 (37.3%)		
Age		65.79 ± 11.1	65.5 [60-72.5]	18-102	65.49 ± 13.59	67.5 [61.75-73]	15-89	.442
Female#			19 (13.8%)			10 (12.2%)		.739
Male#			119 (86.2%)			72 (87.8%)		
Etiology#	HBV		79 (57.3%)			51 (62.2%)		**.006**
	HCV		34 (24.7%)**			6 (7.3%)		
	HDV		2 (1.4%)			0 (0.0%)		
	Cryptogenic		9 (6.5%)			10 (12.2%)		
	NAFLD		14 (10.1%)			15 (18.3%)		
AST		89.3 ± 103.22	60 [38-99.75]	21-941	60.28 ± 52.07	42 [28-75.75]	11-288	**.001**
ALT		51.82 ± 47.58	40 [26-68.25]	11-430	48.44 ± 42.65	33 [20-58.75]	9-232	.104
PLT		158.47 ± 88.65	139 [96.75-186.75]	17-550	253.65 ± 73.38	249 [199.75-305]	116-464	**<.001**
ALB		3.11 ± 0.68	3.06 [2.6-3.6]	1.16-4.67	4.47 ± 5.4	3.64 [3.19-4.1]	1.8-38.9	**<.001**
T. Bil.		1.8±2.21	1.11 [0.86-1.92]	0.49-20	1.46 ± 2.43	0.8 [0.6-1.12]	0.24-17.5	**<.001**
INR		1.37 ± 1.66	1.18 [1.1-1.32]	0.95-20.6	1.11 ± 0.12	1.08 [1.03-1.15]	0.9-1.6	**<.001**
APRI		0.94 ± 2.61	0.44 [0.26-0.75]	0.07-27.68	0.25 ± 0.22	0.17 [0.13-0.31]	0.05-1.27	**<.001**
Survival (months)		25.8 ± 35.91	11.5 [3-32.25]	1-155	30.18 ± 34.48	17.5 [5-45.5]	1-194	**.049**

ALT, alanine transaminase; APRI, aspartate aminotransferase to platelet ratio index; AST, aspartate aminotransaminase; INR, international normalized ratio; ALB, albumin; T. Bil, total bilirubin; NAFLD, non-alcoholic fatty liver disease; HBV, hepatitis B virus; HCV, hepatitis C virus; HDV, hepatitis D virus; SD, standard deviation; IQR, interquartile range.

**Mann–Whitney *U*-test, #Chi-square test.

**Table 2.** Tumor Characteristics and Treatment Choices

		Cirrhotic			Non-cirrhotic			*P*
		Mean ± SD	Median [IQR]	Min-Max	Mean ± SD	Median [IQR]	Min-Max	
MTD#		6.88 ± 4.22	6 [3.5-9.38]	1-19	7.96 ± 5.2	7 [4-10.5]	1.8-24	.227
Tumor focality								**.012**
	1		66 (47.9%)			50 (60.9%)		
	2		7 (5.0%)			1 (1.3%)		
	3		0 (0.0%)			2 (2.5%)		
	>3		35 (25.3%)			23 (28.0%)		
	Infiltrative		30 (21.8%)**			6 (7.3%)		
Portal invasion	Yes		49 (35.3%)			17 (20.7%)		**.021**
AFP#		15 095.57 ± 36 432.02	160 [8.5-6033]	1-291 495	11 724.92 ± 39 672.79	57.15 [4.42-1738]	0-246.362	.188
Treatments	Resection		6 (4.3%)			21 (25.6%)		**<.001**
	Tx		13 (9.4%)			7 (8.5%)		.999*
	Ablation		4 (2.9%)			5 (6.1%)		.247*
	TACE		19 (13.7%)			9 (11.0%)		.548
	TARE		54 (38.8%)			68 (82.9%)		**<.001**
	Systemic theraphy		29 (20.9%)			15 (18.3%)		.626
	Supportive care		41 (29.5%)			11 (13.4%)		**.006**

MTD, maximal tumor diameter/cm; AFP, alfa-fetoprotein; TACE, transcatheter arterial chemoembolization; TARE, transarterial radioembolization; Tx, transplantation; SD, standard deviation; IQR, interquartile range.

#Mann–Whitney *U* test, *Chi-square test, **Fisher’s exact test.

**Table 3.** Factors Affecting Survival in Cirrhotic Patients

Cirrhotic	Univariate		Multivariate	
	Exp (B) (95% CI)	*P*	Exp (B) (95% CI)	*P*
Age	0.978 (0.962-0.994)	**.009**	0.973 (0.953-0.993)	**.009**
Sex (female)	0.736 (0.411-1.319)	.304	0.786 (0.39-1.586)	.501
Etiology				
HBV	Ref.			
HCV	0.59 (0.36-0.95)	**.032**	0.834 (0.489-1.423)	.505
HDV	1.11 (0.27-4.56)	.855	1.182 (0.281-4.972)	.819
Cryptogenic	0.64 (0.26-1.6)	.339	0.379 (0.119-1.207)	.101
NAFLD	1.5 (0.8-2.79)	.204	2.026 (0.887-4.627)	.094
MTD	1100 (1048-1153)	**<.001**	1.084 (1.024-1.148)	**.006**
Tumor focality				
No	Ref.			
1	0.415 (0.057-3.037)	.386	0.983 (0.29-3.327)	.978
2	0.479 (0.049-4.647)	.526	*	*
>3	0.559 (0.075-4.18)	.521	1.291 (0.757-2.201)	.348
Infiltrative	1.691 (0.228-12.523)	.607	2.13 (1.103-4.114)	**.024**
Portal invasion	2.008 (1.339-3.013)	**.001**	1.218 (0.744-1.996)	.433
AFP	1.000005 (0.999-1.00001)	.063	1.000002 (0.999-1.000008)	.513

Cox regression analysis *unable to calculate.

HBV, hepatitis B virus; HCV, hepatitis C virus; HDV, hepatitis D virus; MTD, maximal tumor diameter/cm; AFP, alfa-fetoprotein; NAFLD, non-alcoholic fatty liver disease.

**Table 4.** Factors Affecting Survival in Non-cirrhotic Patients

Non-cirrhotic	Univariate		Multiple	
	Exp (B) (95% CI)	*P*	Exp (B) (95% CI)	*P*
Age	0.999 (0.978-1.020)	.924	0.999 (0.972-1.027)	.937
Sex (female)	1.161 (0.491-2.748)	.734	1.045 (0.37-2.953)	.933
Etiology				
HBV	Ref.			
HCV	0.26 (0.03-1.93)	.187	0.362 (0.047-2.81)	.331
Cryptogenic	2.33 (1.04-5.21)	**.039**	3.036 (1.305-7.065)	**.010**
NAFLD	2.33 (1.12-4.84)	**.024**	2.023 (0.829-4.939)	.122
MTD	1.025 (0.977-1.075)	.321	0.951 (0.882-1.025)	.186
Tumor focality				
No	Ref.			
1	0.049 (0.01-0.243)	**<.001**	0.765 (0.097-6.045)	.799
2	0.039 (0.003-0.492)	**.012**	*	
3	*		1.735 (0.735-4.095)	.209
>3	0.092 (0.018-0.462)	**.004**	2.77 (0.692-11.099)	.150
Infiltrative	0.2 (0.034-1.187)	.072	*	
Portal invasion	2.323 (1.179-4.579)	**.015**	2.816 (1.1-7.208)	**.031**
AFP	1.000008 (1.000003-1.000 014)	**.002**	1.000009 (1.000 002-1.000 016)	**.009**

Cox regression analysis *unable to calculate.

HBV, hepatitis B virus; HCV, hepatitis C virus; HDV, hepatitis D virus; MTD, maximal tumor diameter/cm; AFP, alfa-fetoprotein; NAFLD, non-alcoholic fatty liver disease.
